# Transcriptome-constrained genome-scale metabolic modeling reveals central carbon and amino acid metabolic reprogramming underlying colistin-sulbactam synergy in *Acinetobacter baumannii*

**DOI:** 10.1128/aac.01848-25

**Published:** 2026-05-05

**Authors:** Xingchen Bian, Yan Zhu, Xiaofen Liu, Xin Li, Wanzhen Li, Jinxin Zhao, Jian Li, Jing Zhang

**Affiliations:** 1 Clinical Pharmacology Research Center, Huashan Hospital, Fudan University 12478, Shanghai, China; 2 Institute of Antibiotics, Huashan Hospital, Fudan University 12478, Shanghai, China; 3 Key Laboratory of Clinical Pharmacology of Antibiotics, National Health Commission of the People's Republic of China, Shanghai, China; 4 National Health Commission & National Clinical Research Center for Aging and Medicine, Huashan Hospital, Fudan University 12478, Shanghai, China; 5 Systems Biology Center, Tianjin Institute of Industrial Biotechnology, Chinese Academy of Sciences 165087 , Tianjin, China; 6 Infection Program and Department of Microbiology, Biomedicine Discovery Institute, Monash University 2541, Melbourne, Victoria, Australia; Shionogi Inc. Florham Park, New Jersey, USA

**Keywords:** carbapenem-resistant *A. baumannii*, constraint-based genome-scale metabolic model, polymyxin, beta-lactam inhibitor, combination therapy, flux variability analysis

## Abstract

This study aimed to construct a genome-scale metabolic model (GSMM) for *Acinetobacter baumannii* and integrate transcriptomic data from colistin, sulbactam, and their combination to delineate the mechanism of synergistic metabolic perturbations. A GSMM of *A. baumannii* 163560 was reconstructed with CarveMe and curated using literature and Biolog assays. Transcriptomic data under different antibiotic treatments were integrated with the model using RIPTiDe, and flux variability analysis was conducted to identify metabolic perturbations across monotherapies and combination therapies. The curated model (2,103 reactions, 1,492 metabolites, and 1,033 genes) achieved prediction accuracies of 80% for gene essentiality and 80.5% for carbon source utilization. Transcriptomic constraints substantially reduced the active network across all treatments while retaining core pathways such as the TCA cycle and amino acid metabolism. Colistin uniquely activated the glyoxylate cycle, consistent with a stress-adaptive bypass of central carbon metabolism. In contrast, the colistin-sulbactam combination induced distinct shifts toward amino acid, nucleotide, and ion metabolism, with aspartate-related pathways showing characteristic flux increases. Flux variation analysis further demonstrated enhanced TCA cycle activity under colistin and combination therapy, likely reflecting an oxidative stress response. Combination treatment also revealed unique essential reactions involving nucleotide and amino acid biosynthesis. Colistin and sulbactam elicit distinct metabolic rewiring in *A. baumannii*. Combination therapy drives a shift from central carbon metabolism toward amino acid and ion metabolism. Enzymes such as isocitrate lyase, malate synthase, and aspartate deaminase—absent in mammals—represent potential targets to enhance synergistic antibacterial effects.

## INTRODUCTION

*Acinetobacter baumannii* (*A. baumannii*) is a critical pathogen, causing severe nosocomial infections worldwide (1, 2). The suboptimal use of antibiotics has led to a significant increase in carbapenem resistant *A. baumannii* (CRAB) in China, with the detection rate rising from nearly 40% in 2005 to approximately 76% in 2025 (3). The mortality rates of CRAB-related bloodstream infections, central nervous system infections, and ventilator-associated pneumonia reach 50%–73.6% (4, 5). Even with the approval of several new beta-lactam/beta-lactam inhibitors, in middle-to-low-income countries, polymyxins (i.e., colistin and polymyxin B) are used as a last-line therapy against CRAB, with a susceptibility rate exceeding 90% (3). However, polymyxin monotherapy is limited by nephrotoxicity (33%–60.4% incidence of acute kidney injury) and heterogeneous resistance (rates up to 83%) (6, 7). Therefore, combination therapy is widely recommended for treating CRAB infections in clinical practice (8).

A retrospective study indicated that colistin/carbapenems and colistin/sulbactam combinations for extensively drug-resistant *A. baumannii* did not reveal significant differences with respect to 14-day survival and clinical or microbiological outcomes (9). Complete response/cure and 14-day survival were relatively higher (46.3% vs. 30.6% and 68.2% vs. 55.5%, respectively), and microbiological eradication was significantly higher in the combination group (79.9% vs. 55.6%, *P* = 0.001) (9). A systematic review and meta-analysis on 2,529 patients from 29 studies concluded that colistin combined with sulbactam was safer and more efficacious than colistin monotherapy against CRAB-associated infections (10). A recent propensity score analysis indicated that combining colistin and sulbactam effectively reduced mortality in 7 days in patients with severe CRAB pneumonia (11). A favorable safety profile, together with comparable efficacy to other colistin-based combinations, makes colistin combined with sulbactam an advantageous synergistic option for the treatment of CRAB infections.

Our previous study has demonstrated the synergistic effect of polymyxins combined with sulbactam (12). Transcriptomic analysis revealed that this synergy involves broad metabolic disturbances, primarily affecting cell envelope biogenesis and phospholipid metabolism (13). Colistin rapidly disrupted bacterial membranes and fatty acid metabolism, while the combination intensified these effects, further enhancing cell wall and phospholipid synthesis. Metabolomic profiling also showed that the combination more strongly depleted amino acid and nucleotide pools than either monotherapy (14).

Increasing evidence indicates that antibiotic efficacy depends not only on target binding but also on dynamic interactions with bacterial metabolism (15). Antibiotics reshape metabolic fluxes, while adaptive metabolism modulates antibiotic activity. Since transcriptomics and metabolomics alone cannot reveal flux directionality, genome-scale metabolic models (GSMMs) provide a powerful framework to quantify pathway activity and interpret antibiotic mechanisms. Several strain-specific GSMMs have been established for *A. baumannii*—including iCN718, iLP844/iATCC19606, and iAB5075—enabling system-level exploration of metabolic rewiring under antimicrobial stress (16–18). Recent modeling workflows (e.g., CarveMe [19], AuReMe [20], and RAVEN Toolbox [21]) and contextualization algorithms (e.g., GIMME, iMAT, and RIPTiDe [22]) integrate omics data to identify condition-specific flux changes and predict synergistic drug interactions (23).

In the representative study, we established a GSMM of an MDR clinical *A. baumannii* isolate from a bloodstream infection, which demonstrated a synergistic effect of the colistin-sulbactam combination. This model was used to illustrate the dynamic metabolic responses to colistin and sulbactam, both individually and in combination, under transcriptomic constraints. The contextualized models predicted that essential genes or specific reactions shift from central carbon metabolism under colistin treatment alone to amino acid and nucleotide metabolism under colistin–sulbactam treatment. This study refines our understanding of the role of the TCA cycle in the mechanisms of action of colistin and sulbactam through systems pharmacology approaches and proposes putative drug targets to enhance the efficacy of colistin.

## MATERIALS AND METHODS

### Construction of a genome-scale metabolic model

*A. baumannii* 163560 was selected based on our prior *in vitro* PK/PD and transcriptomic studies demonstrating synergistic activity between colistin and sulbactam in this clinical isolate (12, 13). The draft model of strain 163560 (Genbank accession number GCF_014560305.1) was initially constructed using the automatic tool CarveMe, gap-filled, and initialized with LB media (19). Extensive manual curation was conducted using substrate utilization phenotypes derived from Biolog phenotypic microarray (24). The model quality was evaluated by Memote to identify blocked reactions (reactions incapable of carrying flux) or dead-end metabolites (metabolites that are only consumed or only produced) (25, 26).

### Gene essentiality

The predictions of essential genes were conducted using COBRApy (27). *In silico* single-gene deletion was conducted, and the genes whose deletion caused bacterial growth rate less than 0.01 h^−1^ in Luria-Bertani were considered as essential. The lists of essential genes of *A. baumannii* ATCC17978 and AB5075-UW were chosen as reference. These essential genes were determined through a genome-wide transposon mutant library analysis (28, 29). Reciprocal BLASTp was conducted using TBtools to get the orthologs between strain 163560 and ATCC17978, or 163560 and AB5075-UW (30). If a gene is essential in ATCC17978 or AB5075-UW, and this gene has a homologous sequence in 163560, then the corresponding gene in 163560 is considered truly essential. The criteria for determining orthologs are sequence identity >70%, E value <1 × 10^−5^, and coverage >70%.

The model-predicted results were compared with the essential genes from homologous sequence analysis. If both methods determine the gene as essential or non-essential, the prediction is considered a true positive or true negative. The accuracy of essential gene prediction is calculated as the sum of true-positive and true-negative genes divided by the total number of genes in the model.

### Biolog phenotype microarray

Biolog phenotypic microarray was conducted to test *A. baumannii* 163560 growth in each single carbon source from PM1 and PM2. Cellular respiration was monitored using Biolog’s tetrazolium-based redox dye, which enhances the detection signal associated with NADH production. The overnight culture was adjusted to OD_590_ 0.170 and diluted with the mixture of growth media IF-0 (mainly consists of inorganic salts and trace elements) and dye to the final OD_590_ 0.032. The absorbance at 595 nm was evaluated after incubation at 35°C for 24 h. The ratio of OD595 in a single carbon source to OD595 without a carbon source greater than 1.5 was defined as growth.

### Predictions of carbon source utilization

Flux balance analysis (FBA) was applied to predict bacterial growth in each single carbon source. The flux for each single carbon source in the model was set to a range of −10 to 0 mmol·gDW⁻¹·h⁻¹ sequentially, where negative values indicate that the substance can be consumed. The maximum bacterial growth rate on a single carbon source was optimized under the objective function and mathematical constraints below:


$$maxv_{biomass}$$

s.t.S∙v=0

$$a_{j}\leq v_{j}\leq b_{j},j=1,2\ldots n$$

***S*** represents the stoichiometric matrix of metabolites and metabolic reactions, which conforms to mass balance, v_j_ (mmol·gDW⁻¹·h⁻¹) represents the reaction flux with its upper and lower bounds to be b_j_ and a_j_. If the growth rate is < 0.01 h⁻¹, the carbon source is considered non-utilizable for growth; otherwise, it is considered utilizable. By comparing model predictions with Biolog experimental results, the predictions were labeled as true positive (TP), true negative (TN), false positive (FP), and false negative (FN). The accuracy of the model predictions was calculated as (TP+TN)/(TP+TN + FP+FN). Metabolites and metabolic reactions were removed or added if they were labeled as false positive or false negative, respectively. The model was named LB163560_final after manual curations.

### Contextualized modeling with transcriptomic data

To systemically illustrate the modes of actions of colistin monotherapy and in combination with sulbactam, the gene expression abundance from RNA sequencing (13) was integrated with the model LB163560_final with python package RIPTiDe, which integrates transcript abundance by weighting reactions according to the full expression distribution and applying a parsimonious flux minimization framework, thereby avoiding arbitrary expression thresholds required by methods such as GIMME or iMAT (22). RIPTiDe formulates the problem as a constrained optimization that identifies the most transcriptionally consistent flux state while maintaining network feasibility. This approach was well suited to our single-condition transcriptomic data set and allowed us to generate a condition-specific model without extensive parameter tuning. The MICs of colistin and sulbactam against *A. baumannii* 163560 were 1 mg/L and 32 mg/L, respectively. For the transcriptomic experiments, bacteria were exposed to 2× MIC concentrations of colistin and sulbactam (inoculum density: 10^8^ CFU/mL) for 4 h prior to RNA extraction. The Flux sampling was then performed with the Gapsplit sampler using standard parameters and 500 samples (31). Flux variability analysis for the constrained models was also performed to establish flux ranges for each reaction (32).

Essential genes were predicted using constraint-based models. Based on 500 flux sampling results, the Wilcoxon signed-rank test was used to identify whether there was a significant flux alteration under drug treatment, with significance defined as a BH-corrected *P*-value < 0.05. For reactions with absolute flux values >0.1 and significant flux change with drug treatment, metabolic pathway analysis was conducted using databases such as BiGG and Biocyc. Adobe Illustrator CC 2018 was used for the visualization of metabolic pathways.

### Integrative analysis with metabolomic data

Metabolic state analysis can follow two approaches. A reaction-centered method uses reaction fluxes to reflect metabolic conditions. Alternatively, a metabolite-centered method, using flux-sum calculations, accounts for how metabolite abundance changes due to production or consumption. Metabolomic data from *A. baumannii* 090342 with colistin (MIC = 0.5 mg/L) and sulbactam (MIC = 128 mg/L) treatment were used for comparison (14). Metabolomic profiling was performed under exposure to 2× MIC colistin in combination with 1× MIC sulbactam for 4 h. Common metabolites that were identified in both metabolomics and the metabolic model were chosen for further analysis. The flux sum was calculated as *φ_i_* = 0.5∑|*S_ij_*|·|*v_j_*|. *φ_i_* represented flux sum of metabolite i, *S_ij_* represented the stoichiometric matrix, and *v_j_* was metabolic flux. Decreased abundance and flux sum suggest reduced production. Decreased abundance but increased flux sum suggests increased consumption. Increased abundance and decreased flux sum suggest reduced consumption. Increased abundance and flux sum suggest enhanced production.

## RESULTS

### Gene essentiality: predictions of carbon source utilization

To evaluate the predictive performance of the reconstructed model, *in silico* single-gene deletion analysis and Biolog phenotype microarray assays were performed for gene essentiality and carbon source utilization, respectively. The genome of strain 163560 contains 3,647 coding proteins. The draft model, LB163560_draft, includes 1,033 genes, 1,491 metabolites, and 2,228 metabolic reactions, with a growth rate of 1.91 h⁻¹. Memote testing showed that the model contains no dead-end metabolites and includes 106 blocked reactions (4.80%), which have been manually removed. After optimization based on Biolog experimental results, the final model, LB163560_final, retains 1,033 genes, 1,492 metabolites, and has a reduced number of metabolic reactions, now totaling 2,209. The growth rate decreased to 1.64 h⁻¹. Comprehensive reaction information, including reaction ID, reaction name, reaction formulas, substrates, products, stoichiometries, subcellular compartments, and gene-protein-reaction (GPR) associations, is provided in Table S1. Reactions without GPR represent non-enzymatic or gap-filled reactions.

The BLASTp results show that out of the 3,647 coding proteins in strain 163560, 3,273 (89.7%) are homologous to proteins in AB5075-UW. AB5075-UW has 438 essential genes, and based on the homology alignment, strain 163560 has 402 essential genes. Similarly, 3,369 proteins (92.4%) in strain 163560 are homologous to proteins in ATCC17978. ATCC 17978 has 453 essential genes, with strain 163560 having 435 essential genes based on alignment. The essential genes identified in strain 163560 were compared to model predictions, as shown in Table 1. The prediction accuracy of essential genes, using AB5075-UW and ATCC17978 as references, is 82.8% and 78.8%, respectively. A comparison between model-predicted and Biolog experimentally determined single carbon source utilization is provided in Table S2. Based on the Biolog assay, the predictive accuracy of single carbon source utilization of the draft model was 67.9% and was improved to 80.5% via deleting 22 false-positive metabolites and adding 2 false-negative metabolites and reactions (Table 1 and Tables S3 and S4).

**TABLE 1:** Predictive performance of essential genes and carbon source utilization^*a*^

Prediction result	Essential genes		Carbon source utilization
	AB5075-UW	ATCC17978	LB163560_final
True positive	75	73	27
True negative	780	740	126
False positive	22	24	0
False negative	155	195	37
Accuracy	82.8%	78.8%	80.5%

^*a*^ Model-predicted essential genes were evaluated against ortholog-based essential gene data sets from strains AB5075-UW and ATCC17978. Carbon source utilization predictions were validated using Biolog phenotypic microarray assays.

### Contextualized modeling using transcriptomic data

Transcriptomic data under each antibiotic condition were integrated into LB163560_final using RIPTiDe to generate context-specific metabolic models. The numbers of genes, metabolites, and reactions for each model are shown in Table 2. Average decreases in the number of model reactions, metabolites, genes, and growth rates were about 85%, 77%, 66%, and 73% with transcript constraint. Among the four constrained models, 317 core metabolic reactions were identified. Reactions exclusively in a given treatment-specific model were identified to characterize antibiotic-specific metabolic rewiring. Eleven unique metabolic reactions were identified with colistin treatment, while 10 were identified with colistin and sulbactam combined treatment group, three of which overlap with the colistin group. The list of unique reactions is shown in Table 3. Under the action of colistin alone, the unique reactions are associated with the tricarboxylic acid cycle, glyoxylate cycle, and serine metabolism. In the combination group, the unique reactions relate to aspartate, alanine metabolism, and iron ion metabolism. These findings indicate a bacteria metabolic shift from central carbon metabolism to amino acid and ion metabolism when the antibiotic stress changes from colistin alone to the combination of colistin and sulbactam.

**TABLE 2:** Basic characteristics of constraint-based metabolic model^*a*^

	LB163560_final	LB163560_final_CTRL	LB163560_final_CST	LB163560_final_SUL	LB163560_final_COMB
Number of reactions	2,209	330	337	330	332
Number of metabolites	1,492	340	347	340	341
Number of genes	1,033	343	353	345	357
Number of essential genes	97	169	166	164	167
Growth rate (h^−1^)	1.64	0.44	0.44	0.44	0.48

^*a*^ The genome-scale model LB163560_final was contextualized with RNA-seq data using RIPTiDe to generate condition-specific models for untreated control (CTRL), colistin (CST), sulbactam (SUL), and combination therapy (COMB). Model size and predicted growth rates are shown.

**TABLE 3:** Treatment-specific metabolic reactions uniquely activated under colistin monotherapy or colistin–sulbactam combination therapy^*a*^

	Metabolic flux (mmol/gDW/h)	Descriptive name	Reactions
LB163560_final_CST			
**ALCD2ir**	**0.10**	**Alcohol dehydrogenase reverse rxn acetaldehyde ethanol**	**acald_c + h_c + nadh_c ⇌ etoh_c + nad_c**
ETOHtex	−0.10	Ethanol transport via diffusion (extracellular to periplasm)	etoh_e ⇌ etoh_p
ETOHtrpp	−0.10	Ethanol reversible transport via diffusion (periplasm)	etoh_p ⇌ etoh_c
ICDHyr	−0.22	Isocitrate dehydrogenase (NADP)	icit_c + nadp_c ⇌ akg_c + co2_c + nadph_c
**ICL**	**0.22**	**Isocitrate lyase**	**icit_c ⇌ glx_c + succ_c**
**MALS**	**0.22**	**Malate synthase**	**accoa_c + glx_c + H2O_c ⇌ coa_c + h_c + mal__L_c**
PGCD	0.15	Phosphoglycerate dehydrogenase	3pg_c + nad_c ⇌ 3php_c + h_c + nadh_c
**PGM_1**	**−0.21**	**Phosphoglycerate mutase**	**2pg_c ⇌ 3pg_c**
PSERT	0.15	Phosphoserine transaminase	3php_c + glu__L_c ⇌ akg_c + pser__L_c
PSP_L	0.15	Phosphoserine phosphatase (L-serine)	H2O_c + pser__L_c ⇌ pi_c + ser__L_c
EX_etoh_e	0.10	Ethanol exchange	etoh_e ⇌
LB163560_final_COMB			
ALDD2x	0.27	Aldehyde dehydrogenase (acetaldehyde, NAD)	acald_c + H2O_c + nad_c ⇌ ac_c + 2.0 h_c + nadh_c
**ASPT**	**0.91**	**L-aspartase**	**asp__L_c ⇌ fum_c + nh4_c**
FE2abc	0.00	Iron (II) transport via ABC system	atp_c + H2O_c + fe2_e ⇌ adp_c + fe2_c + h_c + pi_c
**FGLU**	**0.16**	**Formimidoylglutamase**	**h_c + H2O_c + forglu_c ⇌ glu__L_c + frmd_c**
FORAMD	0.16	FORAMD	H2O_c + frmd_c ⇌ for_c + nh4_c
H2Otpp	−0.05	H2O transport via diffusion (periplasm)	H2O_p ⇌ H2O_c
HISDr	0.16	Histidase r	his__L_c ⇌ nh4_c + urcan_c
IZPN	0.16	Imidazolonepropionase	H2O_c + 4izp_c ⇌ h_c + forglu_c
**URCN**	**0.16**	**Urocanase**	**H2O_c + urcan_c ⇌ 4izp_c**
EX_fe2_e	0.00	Fe2+ exchange	fe2_e ⇌

^*a*^ Reactions exclusively identified in the CST or COMB contextualized models are listed. Flux values represent median simulated fluxes (mmol/gDW/h). Essential reactions are highlighted in bold.

Most of the treatment-specific metabolic reactions are catalyzed by proteins encoded by essential genes. With colistin treatment, enzymes encoded by essential genes include phosphoglycerate mutase, malate synthase, isocitrate lyase, and ethanol dehydrogenase. Phosphoglycerate mutase catalyzes the interconversion of 2-phosphoglycerate and 3-phosphoglycerate in glycolysis. Malate synthase and isocitrate lyase are key enzymes of the glyoxylate cycle: isocitrate lyase cleaves isocitrate into succinate and glyoxylate, after which malate synthase condenses glyoxylate with acetyl-CoA to form malate. In another colistin-specific essential gene prediction, two out of four essential genes encode malonate decarboxylase (epsilon subunit) and argininosuccinate synthase (17). The metabolic products, including acetyl-CoA and fumarate, are intermediates of the TCA cycle and can influence energy metabolism. In the reactions unique to colistin and sulbactam combination, the essential genes include those encoding formamidohydrolase, aspartate deaminase, and uridine phosphorylase, which indicates that essential genes shift from central carbon metabolism to amino acid and nucleotide metabolism under colistin-sulbactam treatment.

**Fig 1:**
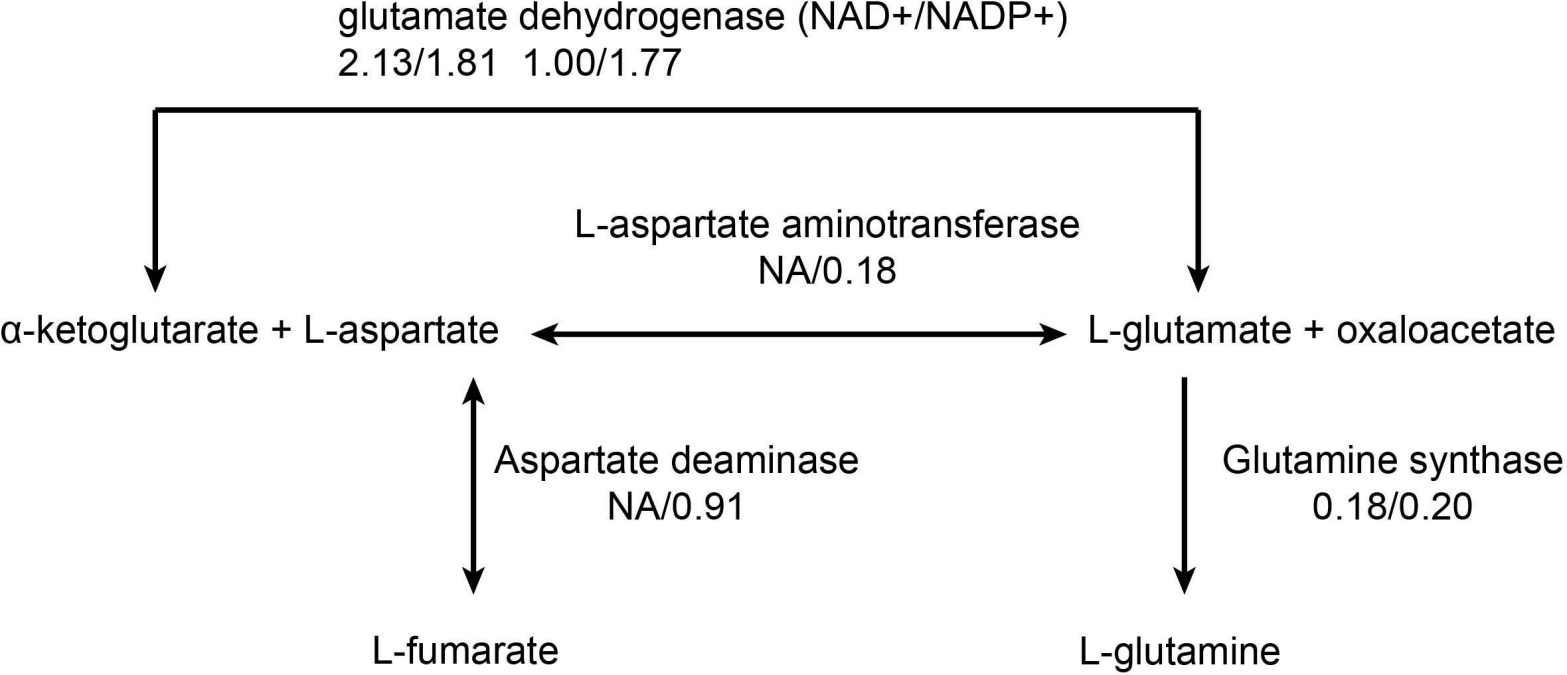
Metabolic flux of aspartate and glutamine metabolic pathways under colistin and sulbactam combined treatment. The number below the enzyme represents the fluxes of the control/combination group (unit: mmol/gDW/h).

Differential flux analysis was performed based on flux sampling results, and significantly, altered reactions were identified using a Wilcoxon signed-rank test with BH-corrected FDR < 0.05 and an absolute flux threshold of 0.1 mmol/gDW/h. With colistin or sulbactam monotherapy or combination therapy, 46, 22, and 60 metabolic reactions, respectively, carried significantly altered fluxes (FDR < 0.05). The primary functions involved include carbohydrate metabolism, energy metabolism, and amino acid metabolism. Comparative analysis of significantly altered flux reactions among each group showed that the three groups share 15 core reactions, primarily enriched in amino acid transport via proton symport and metabolism (Table 4). Under the combined action of colistin and sulbactam, there are 26 unique differential reactions, while under the action of colistin alone, there are five unique differential metabolic reactions. In the unique differential reactions of the combination group, significant changes occurred in the TCA cycle, aspartate metabolism, and glutamine metabolism pathways (Table 5, Fig. 1). In the TCA cycle, α-ketoglutarate and L-aspartate are converted into L-glutamate and oxaloacetate by L-aspartate aminotransferase (NA/0.18 mmol/gDW/h), and L-glutamate is further converted into L-glutamine by glutamine synthetase (0.18/0.20 mmol/gDW/h). L-aspartate is also converted into fumarate by aspartate deaminase (NA/0.91 mmol/gDW/h), with oxaloacetate and fumarate participating in the TCA cycle. L-aspartate is also involved in the biosynthesis of L-lysine, with aspartokinase and aspartate-semialdehyde dehydrogenase catalyzing the first two steps of L-lysine synthesis (Table 5). Additionally, in the fatty acid synthesis process, the conversion of acetyl-CoA to malonyl-CoA, catalyzed by acetyl-CoA carboxylase (0.58/0.62 mmol/gDW/h), and the subsequent conversion of malonyl-CoA to malonyl-ACP (acyl carrier protein, 0.58/0.62 mmol/gDW/h) showed upregulated flux in the combination group (Table 5). Overall, colistin combined with sulbactam not only kills bacteria by disrupting the membrane but also triggers systemic reprogramming of cellular energy metabolism, amino acid (especially aspartate) and lipid biosynthesis, nucleotide metabolism, and transmembrane transport, ultimately leading to a collapse of overall cellular metabolism.

**TABLE 4:** Core metabolic reactions with significantly altered fluxes for colistin, sulbactam, and combination group^*a*^

BiGG ID	Flux_control (mmol/gDW/h)	Flux_combination (mmol/gDW/h)	Flux_colistin (mmol/gDW/h)	Flux_sulbactam (mmol/gDW/h)	Reactions	Descriptive name
ALAt2r	0.13	0.23	0.21	0.22	ala__L_e + h_e ⇌ ala__L_c + h_c	L-alanine reversible transport via proton symport
ASPt2r	0.24	1.34	0.55	0.41	asp__L_e + h_e ⇌ asp__L_c + h_c	L-aspartate reversible transport via proton symport
GLUDxi	2.13	1.81	2.35	2.33	glu__L_c + H2O_c + nad_c --> akg_c + h_c + nadh_c + nh4_c	Glutamate dehydrogenase NAD
GLUDy	1.00	1.77	1.69	1.10	glu__L_c + H2O_c + nadp_c ⇌ akg_c + h_c + nadph_c + nh4_c	Glutamate dehydrogenase (NADP)
MALt2r	−3.05	−4.40	−4.17	−3.35	h_e + mal__L_e ⇌ h_c + mal__L_c	L malate reversible transport via proton symport
NH4t	−3.16	−4.69	−3.93	−3.47	nh4_e ⇌ nh4_c	Ammonia reversible transport
P5CD	0.83	0.73	0.00	0.76	1pyr5c_c + 2 H2O_c + nad_c --> glu__L_c + h_c + nadh_c	1-pyrroline-5-carboxylate dehydrogenase
PROD2	0.83	0.73	0.00	0.76	fad_c + pro__L_c --> 1pyr5c_c + fadh2_c + h_c	Proline dehydrogenase
PROt4	0.88	0.78	0.05	0.81	na1_e + pro__L_e --> na1_c + pro__L_c	Na+/Proline-L symporter
SUCDi	1.21	3.27	3.07	2.24	q8_c + succ_c --> fum_c + q8 h2_c	Succinate dehydrogenase (irreversible)
EX_ala__L_e	−0.13	−0.23	−0.21	−0.22	ala__L_e ⇌	R_EX_ala__L_e
EX_nh4_e	3.16	4.69	3.93	3.47	nh4_e ⇌	R_EX_nh4_e
EX_mal__L_e	3.05	4.40	4.17	3.35	mal__L_e -->	R_EX_mal__L_e
EX_asp__L_e	−0.24	−1.34	−0.55	−0.41	asp__L_e ⇌	R_EX_asp__L_e
EX_pro__L_e	−0.88	−0.78	0.05	−0.81	pro__L_e ⇌	R_EX_pro__L_e

^*a*^ Reactions showing significant flux changes (FDR < 0.05) with absolute flux values > 0.1 mmol/gDW/h in all treatment models are presented. Flux values correspond to median sampled fluxes under each condition.

**TABLE 5:** Unique metabolic reactions with significantly altered fluxes for combination group^*a*^

LB163560_final_COMB	Flux_control (mmol/gDW/h)	Flux_combination (mmol/gDW/h)	Reaction	Descriptive name
ACCOAC	0.58	0.62	accoa_c + atp_c + hco3_c ⇌ adp_c + h_c + malcoa_c + pi_c	Acetyl-CoA carboxylase
ADK1	0.24	0.26	amp_c + atp_c ⇌ 2.0 adp_c	Adenylate kinase
ASAD	−0.13	−0.14	aspsa_c + nadp_c + pi_c ⇌ 4pasp_c + h_c + nadph_c	Aspartate-semialdehyde dehydrogenase
ASPK	0.13	0.14	asp__L_c + atp_c ⇌ 4pasp_c + adp_c	Aspartate kinase
ATPS4rpp	12.66	13.99	adp_c + pi_c + 4.0 h_p ⇌ atp_c + 3.0 h_c + H2O_c	ATP synthase (four protons for one ATP) (periplasm)
CYTBO3_4pp	8.37	9.97	4.0 h_c + 0.5 o2_c + q8 h2_c ⇌ H2O_c + q8 _c + 4.0 h_p	Cytochrome oxidase bo3 (ubiquinol-8: four protons) (periplasm)
GLNS	0.18	0.20	atp_c + glu__L_c + nh4_c ⇌ adp_c + gln__L_c + h_c + pi_c	Glutamine synthetase
HCO3E	0.58	0.62	co2_c + H2O_c ⇌ h_c + hco3_c	HCO3 equilibration reaction
LEUt2r	0.11	0.12	h_e + leu__L_e ⇌ h_c + leu__L_c	L-leucine reversible transport via proton symport
MCOATA	0.58	0.62	ACP_c + malcoa_c ⇌ coa_c + malACP_c	Malonyl-CoA-ACP transacylase
PIt2r	0.19	0.20	h_e + pi_e ⇌ h_c + pi_c	Phosphate reversible transport via proton symport
PPA	0.45	0.49	H2O_c + ppi_c ⇌ h_c + 2.0 pi_c	Inorganic diphosphatase
PRPPS	0.14	0.15	atp_c + r5 p_c ⇌ amp_c + h_c + prpp_c	Phosphoribosylpyrophosphate synthetase
SUCD4	0.83	0.73	fadh2_c + q8_c ⇌ fad_c + q8 h2_c	Succinate dehydrogenase
VALt2r	0.10	0.11	h_e + val__L_e ⇌ h_c + val__L_c	L-valine reversible transport via proton symport
EX_leu__L_e	−0.11	−0.12	leu__L_e ⇌	R_EX_leu__L_e
EX_pi_e	−0.19	−0.20	pi_e ⇌	R_EX_pi_e
EX_val__L_e	−0.10	−0.11	val__L_e ⇌	R_EX_val__L_e
GARFT	0.00	−0.16	10fthf_c + gar_c ⇌ fgam_c + h_c + thf_c	Phosphoribosylglycinamide formyltransferase
GART	0.00	0.16	atp_c + for_c + gar_c ⇌ adp_c + fgam_c + h_c + pi_c	GAR transformylase-T
HISt2r	0.02	0.19	h_e + his__L_e ⇌ h_c + his__L_c	L-histidine reversible transport via proton symport
MTHFC	0.00	−0.16	H2O_c + methf_c ⇌ 10fthf_c + h_c	Methenyltetrahydrofolate cyclohydrolase
MTHFD	0.00	−0.16	mlthf_c + nadp_c ⇌ methf_c + nadph_c	Methylenetetrahydrofolate dehydrogenase (NADP)
NDPK2	0.10	0.10	atp_c + udp_c ⇌ adp_c + utp_c	Nucleoside-diphosphate kinase (ATP: UDP)
EX_his__L_e	−0.02	−0.19	his__L_e ⇌	R_EX_his__L_e

^*a*^ Reactions significantly perturbed only in the COMB model (FDR < 0.05, |flux| > 0.1 mmol/gDW/h) are listed, highlighting combination-specific metabolic reprogramming toward amino acid, nucleotide, and energy metabolism.

**Fig 2:**
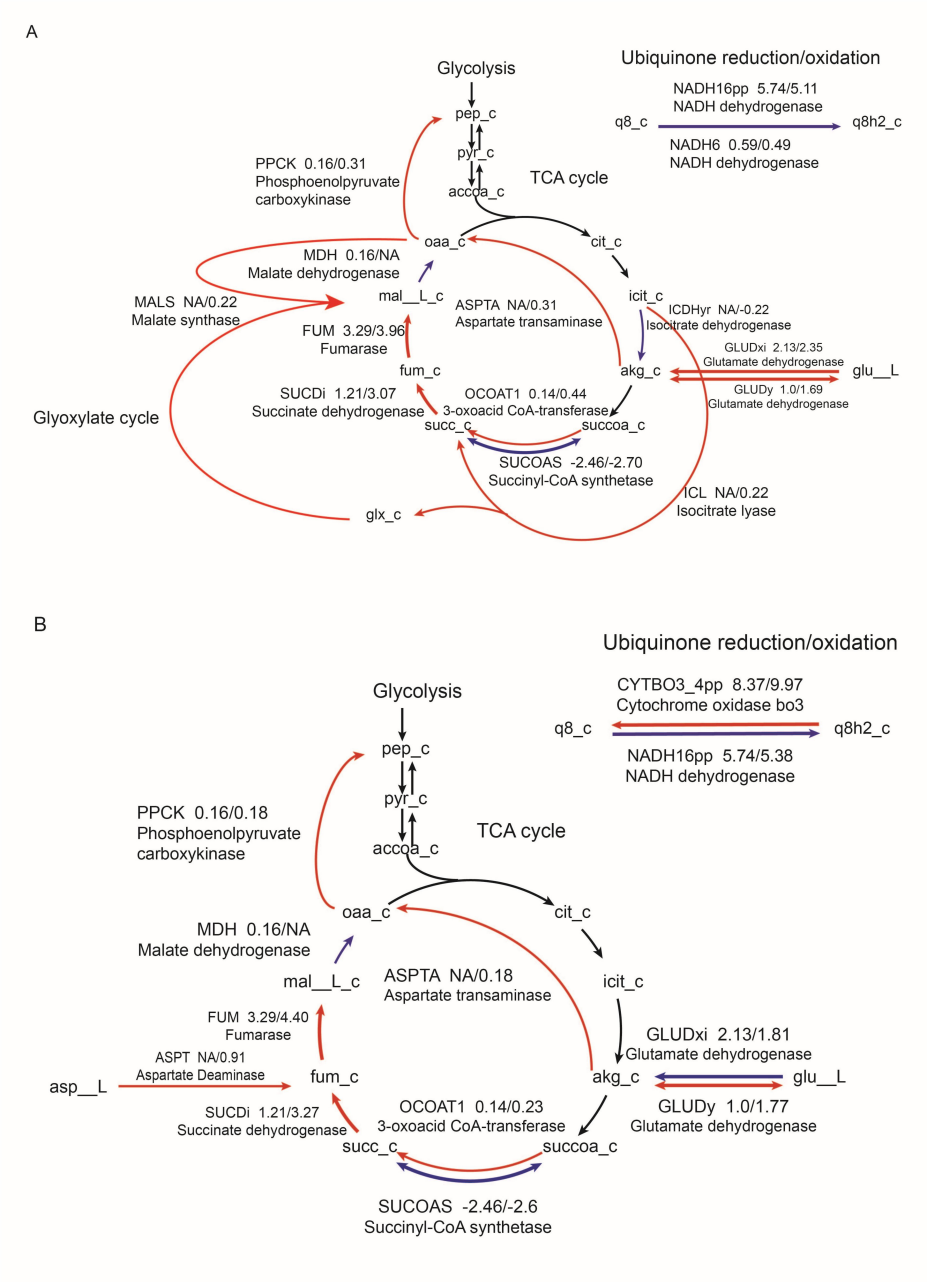
Flux changes in glycolysis, TCA cycle, and ubiquinone oxidation/reduction under colistin monotherapy (**A**) and combination therapy with sulbactam (**B**). Each metabolic reaction label includes its BiGG ID, the flux ratio of the control group to the Colistin group, and the enzyme catalyzing the reaction. Line thickness represents flux magnitude; blue indicates reduced flux, and red indicates increased flux. Due to differences in reactions between the two models, NA denotes reactions not identified in one model. Each node represents a metabolite ID, with "_c" indicating intracellular metabolites. Abbreviations: pep, phosphoenolpyruvate; pyr, pyruvate; accoa, acetyl-CoA; cit, citrate; icit, isocitrate; akg, α-ketoglutarate; succoa, succinyl-CoA; succ, succinate; fum, fumarate; mal, malate; oaa, oxaloacetate; q8, coenzyme Q8; q8h2, reduced coenzyme Q8; glu__L, L-glutamate; asp__L, L-aspartate; glx_c, glyoxylate.

The metabolic fluxes of central carbon metabolism were significantly disturbed with colistin alone and combined with sulbactam, whereas the stress response in glyoxylate shunt (bypass of TCA cycle) was more pronounced with colistin alone. Figure 2 shows the changes in flux for partial glycolysis, TCA cycle, and ubiquinone oxidation/reduction under the effects of colistin alone (A) and in combination with sulbactam (B). It is evident that colistin alone significantly impacts the TCA cycle, with an overall trend of upregulated metabolic flux. The glyoxylate cycle is activated with colistin treatment, indicating a carbon source shift from TCA to the glyoxylate cycle to resist antibiotic pressure. In redox reactions, the reduction flux of ubiquinone-8 decreases under the action of NADH dehydrogenase. In contrast, under the combined treatment, the flux of TCA cycle reactions also shows an upward trend, and in redox reactions, coenzyme Q8 is likely to remain primarily in its oxidized form due to the combined effects of cytochrome *bo3* oxidase and NADH dehydrogenase.

### Integrative analysis of metabolite abundance and metabolic flux

**Fig 3:**
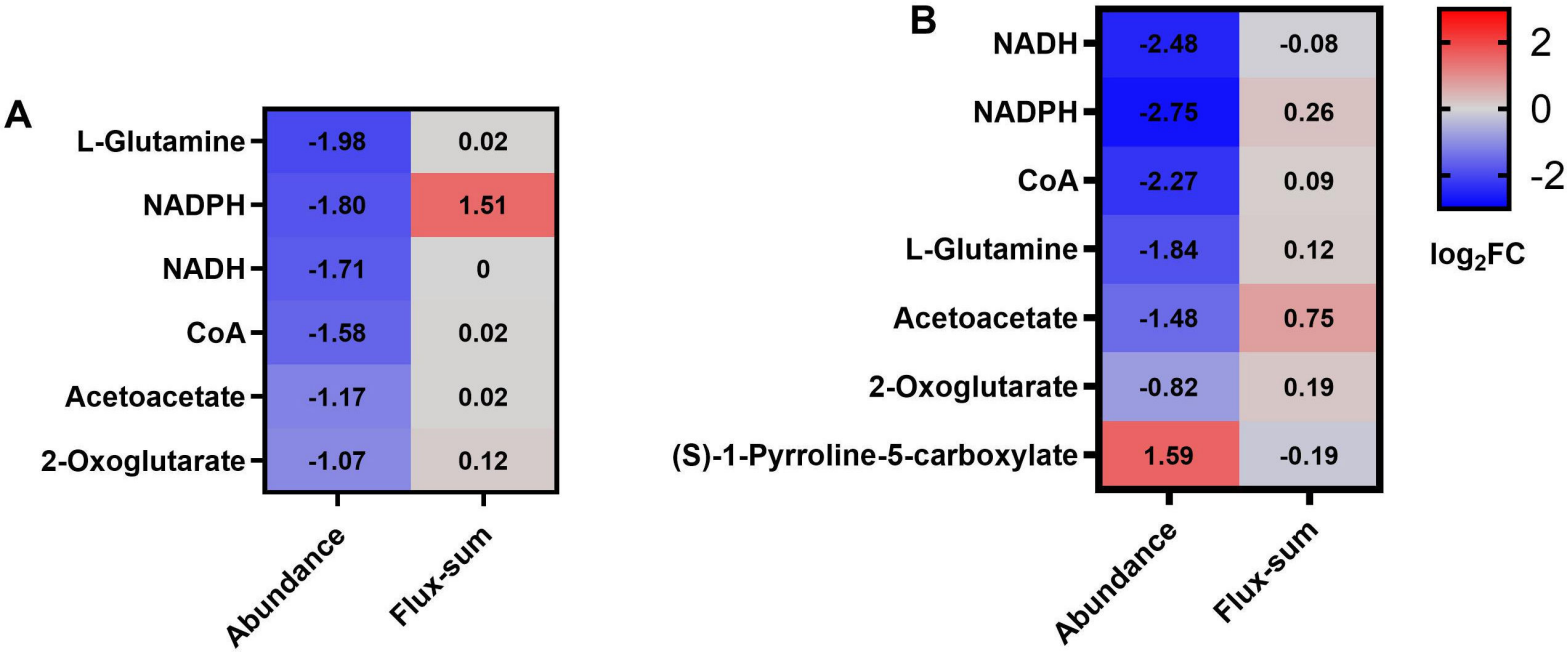
Log_2_ (fold change) of abundance and log_2_ (fold change) flux-sum of metabolites with sulbactam treatment alone (**A**) or in combination with colistin (**B**).

To link metabolite abundance changes with predicted metabolic activity, flux-sum values were calculated for metabolites shared between the metabolomic data set and the GSMM. For the colistin monotherapy group, none of the metabolites with significantly altered abundance identified through metabolomic profiling were mapped to the GSMM model. For the sulbactam monotherapy group, among the 162 differential metabolites identified in metabolomics, 20 were identified in the model, but only 6 had a flux-sum > 0.1 (L-glutamine, NADPH, NADH, CoA, acetoacetate, and 2-oxoglutarate) (Fig. 3). The general trend of decreased metabolite abundance, accompanied by elevated flux activity, suggests an increased metabolic demand and enhanced consumption of intermediates to support stress adaptation and survival. For the combination group, among the 270 differential metabolites identified, 32 were consistent with the model, and 7 had a non-zero flux sum (Fig. 3). Notably, metabolites such as 2-oxoglutarate and L-glutamine showed reduced abundance despite elevated fluxes, implying accelerated carbon and nitrogen flux through the TCA cycle and amino acid metabolism.

## DISCUSSION

Colistin-based regimens, often in combination with sulbactam, have demonstrated efficacy against CRAB. Previous transcriptomic and metabolic studies explored the CRAB response to both antibiotics; however, the metabolic fluxes were still unclear, which may affect our understanding of the synergistic mechanism. In this study, a metabolic model of *A. baumannii* 163560 was constructed and contextualized with transcriptomic data under the effects of colistin and sulbactam, both individually and in combination. The contextualized models predicted that essential genes or specific reactions shift from central carbon metabolism under colistin treatment alone to amino acid, nucleotide, and iron ion metabolism under colistin–sulbactam treatment. The context-specific essential genes, particularly those involved in the central carbon metabolic process, which are absent in mammals, suggest that this pathway could serve as a potential combination target for colistin. This work investigated the mechanisms of action and synergy between the two drugs, providing a reference for identifying new synergistic drug targets.

Increasing evidence indicates that antibiotic efficacy arises not only from target engagement but also from dynamic interactions with bacterial metabolism (13). Antibiotics reshape metabolic fluxes, while adaptive metabolism modulates antibiotic activity. Neither transcriptomics nor metabolomics alone can delineate which fluxes are enhanced or suppressed under antibiotic treatment. Discriminating high- and low-activity pathways is therefore critical for precisely explaining modes of action of antibiotics and optimizing therapeutic strategies. Metabolic flux of the TCA cycle increased under the effect of colistin alone and in combination with sulbactam. This suggests that *A. baumannii* counteracts the oxidative damage caused by colistin alone or in combination with sulbactam by enhancing TCA cycle activity, thereby generating more reducing equivalents NADH and FADH_2_ (33). On the other hand, activation of the TCA cycle may alter the overall physiological state of bacteria to a high metabolic state. Bacteria in such a high metabolic state are generally more susceptible to many antibiotics. For instance, the combination of ATP and meropenem enhances the bactericidal effect against *A. baumannii* (34). Therefore, how to precisely regulate the TCA cycle to enhance bacterial killing warrants further investigation.

Constraint-based modeling, often guided by expression data, has elucidated antibiotic mechanisms, resistance liabilities, and combination effects at the metabolic layer, complementing omics and phenotypic assays. Colistin-induced metabolic alterations are mainly confined to central carbon metabolism and biosynthetic routes, particularly the TCA–glyoxylate axis and serine biosynthesis, indicating a focused metabolic adaptation, and two essential genes under colistin treatment were identified. These essential genes encode isocitrate lyase and malate synthase, which are key enzymes in the glyoxylate cycle. This suggests that inhibiting the activity of isocitrate lyase or malate synthase may lead to synergistic effects with colistin. Studies have shown that the glyoxylate cycle pathway is upregulated in bacterial infections, nutrient deficiency, antibiotic treatments, and bacterial persistence (35, 36). This upregulation could be taken as a stress response while bacteria face unfavorable conditions. Increased glyoxylate cycle activity—particularly through the upregulation of isocitrate lyase—can lead to the accumulation of toxic intermediates that affect bacterial virulence, intracellular infection, and colonization, while also being associated with the activation of multidrug resistance (37, 38). Similarly, increased glyoxylate cycle flux under colistin treatment was predicted in reference strain ATCC19606 (39). Since the glyoxylate pathway is absent in mammals, targeting this pathway for new antimicrobial drug development holds promising prospects. Lead compounds have been developed targeting the highly reactive thiol group of isocitrate lyase cysteine residues, which have shown *in vitro* enzyme inhibitory effects against *Mycobacterium tuberculosis* (40, 41). Notably, activation of the glyoxylate bypass is not exclusive to colistin exposure but represents a conserved adaptive response to diverse environmental and antimicrobial stresses. Therefore, its identification in this study likely reflects a general bacterial stress-associated metabolic program rather than a colistin-specific phenomenon.

Under the combined action of colistin and sulbactam, essential genes and unique reactions shift from central carbon metabolism to amino acid (particularly aspartate metabolism), nucleotide, and iron ion metabolism. The increased metabolic activity in amino acid and nucleotide metabolism was also shown in a metabolomic study (14). The essential gene was found to encode aspartate dehydrogenase, suggesting that interfering with this enzyme’s activity might enhance synergistic bactericidal effects. Serral et al. (42) proposed the top 15 best-ranked metabolic pathways of *K. pneumoniae* according to completeness, number of chokepoints, essentiality, centrality, and human off-targets. The results suggested that under carbapenem treatment, molecules related to energy metabolism, including the TCA cycle, glyoxylate cycle, phenylethylamine degradation, proline degradation, and D-arginine degradation, were attractive targets. In the future, the combination of antimicrobial drugs and metabolic modulators may provide a breakthrough solution to the shortage of effective antimicrobial drugs.

This study integrates metabolic models with transcriptomic data to reveal the metabolic regulation under antibiotic treatment and propose new synergistic targets. The use of transcriptomic data offers broad genomic coverage in *A. baumannii*, enabling a more comprehensive understanding of metabolic alterations through GSMM analysis. However, it is essential to recognize that changes in gene expression do not necessarily translate to changes in metabolic activity. Integrating proteomic constraints can further optimize the metabolic model and produce more accurate predictions. Together, this integrative framework provides a systems-level perspective on antibiotic responses, offering valuable insights for the rational design of synergistic therapeutic strategies.

## Data Availability

The raw and processed data of transcriptomic sequencing can be accessed under accession number GSE218219 in the GEO database.

## SUPPLEMENTAL MATERIAL

Supplemental tables aac.01848-25-s0001.docx Tables S3 and S4.

Table S1 aac.01848-25-s0002.xlsx 2206 reactions of model LB163560_final.

Table S2 aac.01848-25-s0003.xlsx Model-predicted and Biolog-measured utilization of single carbon sources of *A. baumannii* 163560.

ASM does not own the copyrights to Supplemental Material that may be linked to, or accessed through, an article. The authors have granted ASM a non-exclusive, world-wide license to publish the Supplemental Material files. Please contact the corresponding author directly for reuse.
