# Supplementary material for: Transcriptome-constrained genome-scale metabolic modeling reveals central carbon and amino acid metabolic reprogramming underlying colistin-sulbactam synergy in Acinetobacter baumannii
Source: Antimicrob Agents Chemother. 2026 May 5;70(6):e01848-25. doi: 10.1128/aac.01848-25 (PMC13231871; doi:10.1128/aac.01848-25)
Supplement: Supplemental tables — Tables S3 and S4. [file aac.01848-25-s0001.docx]

**Table S3 Deleted false positive exchange reactions**

| **Carbon source** | **BiGG ID** |
| --- | --- |
| D-Galactose | EX_gal_e |
| D-Trehalose | EX_tre_e |
| D-Serine | EX_ser__D_e |
| D-Sorbitol | EX_sbt__D_e |
| Glycerol | EX_glyc_e |
| D-Mannitol | EX_mnl_e |
| L-Rhamnose | EX_rmn_e |
| D-Fructose | EX_fru_e |
| Maltose | EX_malt_e |
| Sucrose | EX_sucr_e |
| Adenosine | EX_adn_e |
| Inosine | EX_ins_e |
| L-Serine | EX_ser__L_e |
| Acetoacetic Acid | EX_acac_e |
| p-Hydroxy Phenyl Acetic Acid | EX_3hoxpac_e |
| 2-Aminoethanol | EX_etha_e |
| Arbutin | EX_arbt_e |
| Salicin | EX_salcn_e |
| Glycine | EX_gly_e |
| L-Isoleucine | EX_ile__L_e |
| L-Lysine | EX_lys__L_e |
| L-Methionine | EX_met__L_e |

Note: The BiGG ID of metabolic reactions was expressed as EX_metabolite ID_e, where EX represents exchange reactions and _e represents extracellular space

**Table S4 Supplemented false negative metabolites and metabolic reactions**

| **Carbon source** | **BiGG ID of metabolite** | **BiGG ID of metabolic reaction** | **Metabolic reaction** |
| --- | --- | --- | --- |
| L-Asparagine | asn__L_e | ASNtex, EX_asn__L_e | asn__L_e ⇌ asn__L_p,  asn__L_e ⇌ |
| Pyruvic Acid | EX_pyr_e | PYRt2rpp | h_p + pyr_p ⇌ h_c + pyr_c |
